# Metallosis mimicking a metabolic disorder: a case report

**DOI:** 10.1016/j.ymgmr.2018.09.005

**Published:** 2018-09-25

**Authors:** Karolina M. Stepien, Zaza Abidin, Graham Lee, Rachel Cullen, Patricia Logan, Gregory M. Pastores

**Affiliations:** a Adult Inherited Metabolic Diseases Department, Salford Royal NHS Foundation Trust, Salford, United Kingdom; b National Centre for Inherited Metabolic Diseases, The Mater Misericordiae University Hospital, Dublin, Ireland; c Clinical Biochemistry and Diagnostic Endocrinology, The Mater Misericordiae University Hospital, Dublin, Ireland; d Ophthalmology Department, The Mater Misericordiae University Hospital, Dublin, Ireland

**Keywords:** Metallosis, Cobalt toxicity, Chromium toxicity, Mitochondrial disease

## Abstract

Metalic prosthesis or occupational exposure are potential sources of systemic cobalt and chromium ion toxicity. The resultant multisystemic clinical presentation can lead to unnecessary investigations before a final etiologic diagnosis is made; with an average delay of a year or more commonly noted.

A 58-year old man presented with cardiomyopathy, pericardial effusion, polycytaemia, polyneuropathy, visual impairment, sudden hearing loss and hypothyroidism over a 2-year period post a metal-on-polyethylene hip replacement surgery.

Biochemistry test results showed serum lactate of 3.8 mmol/L (0.5–2.2 mmol/L). Urine organic acid screen showed mild increases in excretion of tricarboxylic acid cycle intermediates and 2-ethylhydracryllate; suggestive of primary or secondary mitochondrial dysfunction. There were also slight increases in excretion of 4-hydroxyphenyllactate and 4-hydroxyphenylpyruvate suggestive of liver dysfunction. Acylcarnitine profile showed slight increase in hydroxybutyrylcarnitine and tetradeceneoylcarnitine that may reflect ketosis.

In view of his clinical presentation and abnormal metabolic investigations, the initial working diagnosis was mitochondrial disease.

Subsequently, patient presented with hip pain, and radiologic and imaging studies revealed high density collections lateral to the right proximal part of the femur, and medial to the right ilium with signal changes suggestive of metallic content. This prompted toxicology screen which revealed elevated plasma cobalt concentration (903.32 μg/L; reference range: 0.1–0.4) and chromium (71.32 μg/L; <0.5). Six months post right hip prosthesis removal the concentrations have declined and was 61.72 μg/L and chromium 23.97 μg/L. Patient felt some improvement symptomatically, without evident deterioration in his vision or hearing.

This case emphasises careful consideration of past medical history, in patients presenting with multisystemic disease suggestive of mitochondrial dysfunction, and potential causality related to exposure to toxic agents. In retrospect, the absence of a family history could be viewed as a pertinent negative finding. Not uncommonly, specialist focus on their favored system and may not search for a unifying diagnosis. It is likely further delays in diagnosis would have occurred had the patient not developed hip pains, and ultimately referred to the orthopedic surgeons more familiar with similar cases.

## 1 Introduction

Cobalt is a co-factor required for the formation of hydroxycobalamin [1]. Cobalt is transported in circulation by albumin [2], with free fraction estimated to be approximately 5 to 12% [3]. In adults, it is normally stored in the liver and kidneys.

The most common route of exposure is the ion released from metalic prosthesis or occupational exposure (e.g., occupations related to hard metal industry). In surgical patients, the median time from the procedure to first symptoms has been estimated at 19 (3–72) months [4]. Common manifestations include hand tremor, bilateral nerve deafness, sensory-motor polyneuropathy, bilateral optic neuropathy, visual impairment, cognitive decline, retinopathy, cardiomyopathy, pericadial effusion, thyroid toxicity or hypothyroidism, polycythaemia, nausea and weight loss [[4], [5], [6]].

Similarily, exposure to chromium (VI) compounds have been documented to affect respiratory, gastrointestinal, immunological, reproductive and haematological systems [7]. Microcytic hypochromic anaemia and increased white blood cell counts, reticulocytes and plasma haemoglobin were the most common haematological findings [7] and were viewed as indicative of intravascular haemolysis [8,9]. Human exposure to high levels of airborne chromium (VI) in occupational and environmental settings produced symptoms of dizziness, headache, weakness [10] and cerebral oedema [11].

Importantly, metallosis have been shown to mimick skin tumour [12], polymyositis [13], or scrofuloderma [14]. As the clinical presentation of cobalt toxicity is highly non-specific, in the differential diagnosis of such a constellation of symptoms, the existence of mitochondrial genetic defects must be taken into account.

Mitochondrial diseases, however, may present with subtle symptoms also leading to misdiagnosis such as Charcot-Marie Tooth disease [15] or somatoform disorder [16]. In some cases, a positive family history and matrilineal segregation offer valuable clues.

We describe a case of cobalt and chromium toxicity secondary to metal-on-polyethylene hip prosthesis presenting with clinical and biochemical features suggestive of a mitochondrial disorder.

## 2 Case

A 57-year-old male presented to his General Practitioner with 15 months history of progressive fatigue, dyspnoea and numbness over the soles of his feet. Test results showed evidence of hypothyroidism. His chest x-ray showed significant global cardiomegaly, and he was subsequently referred to a cardiologist for the management of his myopericarditis, which necessitated drainage of pericardial effusion. Electrocardiogram showed sinus rhythm rate 80 beats per minute with low voltage complexes in the limb leads and voltage criteria for left ventricular hypertrophy (LVH). Post-drainage echocardiogram appeared characteristic of an infiltrative process. There was moderate concentric LVH with septal hypertrophy (IVSd 1.6 cm) and moderate global hypokinesis of the left ventricle, and an ejection fraction of 25–30%. The right ventricle was moderately dilated, with mildly reduced systolic function. Both atria were mildly dilated. Cardiac MRI showed high normal left ventricular (LV) size with severely increased LV mass, global hypokinesis and moderate hypertrophy with no inflammation or oedema on short-tau inversion recovery (STIR). In addition, there was impaired biventricular systolic function, moderate bi-atrial dilatation, normal native myocardial T1, mildly raised extracellular volume (ECV) 0.36 and diffuse subendocardial late gadolinium enhancement. Moreover, perfusion scan showed severe circumferential inducible ischaemia and transmural fibrosis in the lateral wall at basal and mid ventricular level post-godolinium. An abnormal appearance of the liver and spleen were also noted incidentally; suggestive of iron overload, and there was extensive mediastinal and bi-hilar lymphadenopathy. Coronary angiography, undertaken four months later, showed 70% stenosis over the left anterior descending artery.

These findings were initially attributed to amyloidosis; however, this diagnosis was not supported by further evalautions. Serum amyloid P component (SAP) scintigraphy showed no visceral amyloid uptake. Technetium labelled 3,3-diphosphono-1,2-propanodicarboxylic acid (Tc-DPD) scintigraphy showed no cardiac uptake (ruling out transthyretin cardiac amyloidosis). Additionally studies did not reveal evidence of plasma dyscrasia, and skeletal survey, and bone marrow and trephine biopsy were normal. Ultrasound studies showed diffusely enlarged thyroid glands, thick isthmus with no lymphadenopathy. Fat pad and myocardial biopsies were negative for infiltrative disease; although there were signs of myocyte hypertrophy and patchy fibrosis.

Past medical history revealed perforated right eardrum, two previous varicose veins surgeries and bilateral hip osteoarthritis. He had a ceramic-on-ceramic right total hip replacement 10 years prior to presentation, with revision to a metal-on-polyethylene prosthesis eight years later. Over a period of two years thereafter he began to develope fatigue, poor memory and inattention, increased dizziness and incoordination, nausea, deterioration in vision and hearing, vertigo and tinnitus, pain and numbness over his lower extremities. The complexity of clinical presentation and lack of a unifying diagnosis prompted a referral to the Inherited Metabolic Diseases Unit (IMDU) for investigations. There was no family history of note.

Prior to IMDU referral, patient was also evaluated independently for polycythaemia; and testing was negative for JAK2 mutations. Blood film showed mild persistent eosinophilia with degranulation. RBC (6.30 × 10^12^/L; reference range 4.50–5.50), haemoglobin (189 g/L; 13.0–18.0), and haematocrit (0.572 L/L; 0.400–0.500) were elevated. Autoimmune screen was negative, apart from elevated antinuclear antibody of 320 titre (<80) with a speckled pattern. Anti-double stranded DNA was 10 (elevated). The significance of these findings was uncertain. Evaluations for polyneuropathy also failed to disclose the underlying cause. Nerve conduction studies showed evidence of severe motor-sensory axonal/demyelinating polyneuropathy involving the lower extremities. The upper extremities showed normal conduction velocities with a degree of mild carpal tunnel syndrome on the right.

Physical examination revealed significant peripheral neuropathy, impaired joint-position sense and absent tendon reflexes on his lower limbs. Abdominal examination showed palpable liver and spleen edges with firm consistency. He had hepatosplenomegaly, bilateral sensorineural hearing loss and bilateral visual impairment.

Biochemistry test results showed serum lactate of 3.8 mmol/L (0.5–2.2 mmol/L). Urine organic acid screen showed mild increases in excretion of tricarboxylic acid cycle intermediates and 2-ethylhydracryllate; suggestive of primary or secondary mitochondrial dysfunction. There were also slight increases in excretion of 4-hydroxyphenyllactate and 4-hydroxyphenylpyruvate suggestive of liver dysfunction. Serum amino acid profile was normal. Acylcarnitine profile showed slight increase in hydroxybutyrlcarnitine and tetradeceneoylcarnitine that may reflect ketosis. Serum Brain Natriuretic Peptide was 141 ng/L (<135). Creatine kinase was 61 IU/L (44–272 IU/L) and lactate dehydrogenase was raised at 325 IU/L (120–220 IU/L).

Molecular genetic analysis for suspected mitochondrial disease was undertaken and negative for 3243A > G mutation.

Patient developed further visual problems, which led to a referral to the Ophthalmology Unit. Eye electrophysiology showed poor macular and central retinal function evidenced by absent pattern retinal electrogram (PERG) response, consistent with a mild cone/rod dystrophy or macular dystrophy with mild pan-retinal involvement. There was no evidence of cataract.

Subsequently, he presented to the Emergency Department with severe right sided hip pain and inability to weight bear Right hip X-ray reported high density material surrounding the right hip joint and overlying the right iliac bone, possibly representing extruded orthopedic cement. CT of his right hip showed significant volume of high density homogenous material in the right iliopsoas bursa, anterior to the acetabulum and surrounding the greater trochanter. Small bony bodies and free air were noted. In comparison to the CT scan performed 7 months previously, the observed extrusion into the right iliopsoas bursa was new. Pelvic MRI was performed and confirmed high density collection lateral to the right proximal part of the femur, and medial to the right ilium suggesting metallic content. CT-guided right periprosthetic fluid drainage was undertaken, with 150 ml of black fluid aspirated within the right ilipsoas muscle collection and another 100 ml of black fluid aspirated from the right greater trochanter collection. A total of 300 ml of black fluid was aspirated from around the right hip arthroplasty.

The diagnosis of metallosis was considered. Preoperatively, plasma cobalt concentration (903.32 μg/L; reference range 0.1–0.4) and chromium (71.32 μg/L; <0.5) were found to be markedly elevated. Six months post right hip prosthesis removal the concentrations had declined but were still above the threshold recommended by Medical Health Regulatory Authority; cobalt concentration was 61.72 μg/L and chromium 23.97 μg/L. However, patient reported feeling better, without further deterioration of his vision and peripheral neuropathy. Hearing test showed a severe sensorineural hearing loss that stabilised.

Two years later his repeat plasma lactate, plasma acylcarnitines and urine organic acids were unremarkable.

## 3 Discussion

Mitochondrial disease and metallosis can present with similar clinical and laboratory features, so in the differential diagnosis it is essential to discriminate between these conditions, in relevant clinical settings (e.g., history of exposure to potential toxins) As routine physical and laboratory investigations often provide only indirect evidence as to cause of neuropathy, hearing loss, cardiomyopathy etc., genetic testing may have an essential role in making the correct diagnosis.

Clinical picture and abnormalities in acylcarnitine profile were highly indicative of a mitochondrial disease, ultimately attributed to secondary to long-term cobalt exposure, and which normalized.

### 3.1 Mechanisms of neurotoxicity

Neurotoxicity with resultant demyelination and axonal loss have been previously observed in patients with arthroprosthesis [17,18]. Several mechanisms of cobalt toxicity have been postulated. The mitochondria are the fundamental target of cobalt toxicity, as shown by loss of mitochondrial membrane potential and the apoptogenic factors release from mitochondria [19]. Cobalt as an ionized species Co^2+^ is able to induce the phenomenon of mitochondrial permeability transition in rat mitochondria with the opening of the transition pore. In addition, it may induce mitochondrial swelling, which is prevented by cyclosporin A and other Co^2+^ transport inhibitors and antioxidant agents [20].

Thus, toxic neuropathies may be associated with a disruption in mitochondrial oxidative phosphorylation, similar to mitochondrial neuropathies of genetic and environmental origin [21].

Cobalt has also been shown to be cytotoxic to many cell types, including neural cells [[22], [23], [24]] and it can induce cell death by apoptosis and necrosis [25]. Moreover, it can cause DNA fragmentation [[26], [27], [28]], activation of caspases [25], increased production of reactive oxygen species [26,29,32], augmented phosphorylation of mitogen-activated protein (MAP) kinases [24,25] and transcriptional repression of the human p53 gene [5,29].

Toxic neuropathy can be associated with ototoxicity caused by the production and action of reactive oxygen species on basal outer hair cells that appear to be very sensitive [30]. Reactive oxygen species can deplete cochlear tissues of antioxidant protective molecules, for example, glutathione and antioxidant enzymes [5]. It has been suggested that cobalt mediates the occurrence of oxidative stress which contributes to cell toxicity and death [20]. Cobalt ions generate highly damaging hydroxyl radical and induce oxidation of sulfhydryl groups, glutathione and pyridine nucleotides [20]. It also induces the release of the pro-apoptotic factors, cytochrome *c* and AIF and indices apoptosis with caspase activation and increased level of expression of HIF-1 alpha.

Furthermore, cobaltous ions have been shown to suppress mitochondrial motility causing destruction of axonal mitochondria which precedes the axonal degeneration [31]. In experimental studies, cobalt decreased postsynaptic responses induced by neurotransmiters in vitro and inhibited synaptic transmission through a presynaptic blockage of calcium channels [5,32].

Cobalt-related neurotoxicity has also been shown to be associated with optic nerve and lens changes in rats [32]. The fibres of the optic nerve can be swollen and tortuous with fragmentation in their myelin sheaths. An atrophy of nerve fibres, injury to the ganglion and alteration in the nucleus of photoreceptors in the retina have been demonstracted in experimental study on rabits [[33], [34]].

Neurotoxicity has also been attributed to chromium (VI); which is thought to enter the brain through pathological pallidal blood vessels that showed vascular siderosis and possible breakdown of the blood-brain barrier [35]. In addition, animal studies have shown that chronic chromium exposure may induce hypoactivity in rats [36] and cortical degenerative changes with chromolysis in rabbits given intraperitoneal doses of chromium over a period of 6 weeks [37].

There is an emphasis on an early detection of soft tissue reactions in patients implanted with metal-on-metal hip replacements and the assessment of the process of metalossis in all patients, to permit a balance between the risks of hip surgery and the risks of conservative management in the short term. As specialists tend to focus on their favored system, complex cases may benefit from interdisciplinary interaction, sooner rather than later, on a concerted search for a unifying diagnosis. In this particular case, past medical history of hip replacement and associated complications proved relevant, and serves as a reminder of its carefully consideration.

## Ethics approval and consent to participate

N/A.

## Consent for publication

A consent was obtained from the patient.

## Availability of data and material

N/A.

## Competing interests

Co-authors have no conflict of interest for this paper.

## Funding

N/A.

## Authors' contributions

All co-authors contributed to the manuscript.
